# Necrolytic Migratory Erythema as the First Manifestation of Glucagonoma

**DOI:** 10.1155/2012/974210

**Published:** 2012-08-27

**Authors:** Abolfazl Afsharfard, Khashayar Atqiaee, Saran Lotfollahzadeh, Majid Alborzi, Amir Derakhshanfar

**Affiliations:** Department of General Surgery, Institute of Surgery, Shohaday-e-Tajrish Hospital, Shahid Beheshti University of Medical Sciences, Shahid Chamran Highway Yaman St, Tehran 1985717443, Iran

## Abstract

Necrolytic migratory erythma (NME) as a rare skin disorder that can affected Perineum, distal extremities, lower abdomen and face are the most commonly affected sites.It can be as a part of Glucagonoma syndrome that is defined as an association of glucagonoma with NME, hyperglucagonemia, glucose intolerance, anemia and weight loss. Here, Authors describe a woman admitted to the dermatology ward with NME which was later found to be associated with glucagonoma and multiple hepatic lesions.

## 1. Introduction

Necrolytic migratory erythema (NME) as a rare skin disorder was first described by Becker et al. in 1942 who reported a woman affected by an alpha-cell tumor of pancreas characterized by irregular annular eruption with a serpiginous advancing border [1]. McGavran and coworker reported a case with hyperglucagonemia associated with cutaneous changes [2].

Perineum, distal extremities, lower abdomen, and face are the most commonly affected sites [3, 4] and there is a correlation of alpha-cell pancreatic tumor with a characteristic rash; NME was termed glucagonoma syndrome in 1979 by Mallinson et al. [4]. The syndrome is defined as an association of glucagonoma, NME, hyperglucagonemia, glucose intolerance, anemia, and weight loss [3, 5]. Here, we describe a woman admitted to the dermatology ward with NME, which was later found to be associated with glucagonoma and multiple hepatic lesions.

## 2. Case Presentation

A 53-year-old woman presented with a year history of erosive and bullous lesions of extremities, genitalia, and oral cavity. The erosions progressed to involve the lower extremities, trunk, and upper extremities. At this time, the patient was admitted to the dermatology ward for further evaluation.

The patient was a known case of diabetes mellitus for 11 years with an approximately one-year history of pruritic and painful skin eruptions, treated with topical steroids without clinical improvement, which she was forced to discontinue due to raised blood glucose.

Skin biopsy revealed psoriasiform acanthosis, abrupt necrosis of upper layers of stratum spinosum, and neutrophilic chemotaxis; whereas the lower half of epidermis appears viable, the detached necrolytic portion appears pale with pyknotic nuclei. Also basal layer hyperplasia, vascular change, and a deficient granular layer were noted. Perivascular lymphocytic infiltration and scattered extravasated RBCs were present in the upper dermis, which were all compatible with necrolytic migratory erythema. 

Recognizing the association of these lesions with intraabdominal masses, a computed tomography scan was conducted. The scan confirmed a prominent pancreas tail due to pancreatic mass and a normal liver. Furthermore, a (radio-labeled) octreotide-receptor scintigraphy detected pathologic somatostatin receptor positive mass lesions in the abdomen surrounding the body of pancreas with multiple suspicious small hepatic lesions.

Glucagon level was 2040 ng/L with normal range of 60–177 ng/L. 

Endoscopic ultrasonography discovered a mass lesion (mixed echoic 25–29 mm) at the junction of body and tail of pancreas. Pancreatic head and common bile duct were normal. At this time, a surgical consultation was requested. 

On upper gastrointestinal endoscopy, the only detected pathologic finding was erythematous mucosa of the antrum, which on pathologic assessment was consistent with moderate chronic active gastritis.

Subtotal pancreatectomy and splenectomy was performed. 

Pathologic examination of the mass consisting of gyriform and solid growth pattern of atypical tumoral cells with pale to granular cytoplasms, salt and pepper nuclei, and low mitotic figures embedding in fibritic and focal hyalinized stroma was suggestive of glucagonomas. The spleen specimen showed congestive splenomegaly.

In the days following her operation, the patient was admitted to the Intensive Care Unit, where due to her comorbid conditions she faced different complications including active lower gastrointestinal bleeding, a glutheal abscess, and pulmonary atelectasis, all treated by routine hospital protocols. Eventually the patient developed respiratory failure secondary to acute respiratory distress syndrome and expired 28 days postoperatively. No autopsy was performed. 

## 3. Discussion 

Glucagonoma-producing tumors present a broad spectrum; clinical manifestation varies from asymptomatic to glucagonoma syndrome [5].

Glucagonoma syndrome is defined as an association of glucagonoma with NME, hyperglucagonemia, glucose intolerance, anemia, and weight loss [3, 4]. 

Multiple glucagonomas may be rarely associated with MEN I comprising only 3% of glucagonoma syndrome [6]. 

Association of glucagonoma in MEN I syndrome is remarkable for 80% possibility of malignancy [6].

Diffuse progressive epidermal necrotic rash associated with pancreatic neoplasm was first described by Becker et al. in 1942 [1]. 

More than 20 years later in 1966 McGavran et al. reported a patient who was affected by hyperglucagonemia, eczematoid, erythematous rash, mild diabetes mellitus, anemia, and a glucagon-secreting alpha cell tumor of pancreas simultaneously [2]. 

It was named glucagonoma syndrome [1], while the term necrolytic migratory erythema in order to describe typical skin eruption was primarily called by Wilkinson [3]. 

Based on immunocytochemical findings, glucagonoma is categorized into two subgroups, first the one associated with glucagonoma syndrome and second those not associated with glucagonoma syndrome characterized by multiple small tumors with gyriform microscopic pattern of growth.

In comparison between the two types, the first type presented with a solitary and large tumor and high incidence of malignancy, while the second type is often multiple and small and is always benign [7]. 

Distinct diagnosis of NME is important as it might be the clue for prompt detection of glucagonoma; it may proceed at least one year before a correct diagnosis is made [8]. 

Liver metastasis may be found simultaneously with the primary tumor, which can be possibly detected with somatostatin-receptor scintigraphy with radio-labeled octreotide.

Consideration of diabetes mellitus and a pancreatic tumor (especially in the tail of the pancreas) may be a clue to diagnose an endocrine tumor [9].

Successful palliative treatment with long-acting somatostatin analogues has been reported in the literature [10].

## References

[1] Becker SW, Brennan BB (1961). Benign and malignant cutaneous tumors in the elderly. *Archives of Dermatology*.

[2] McGavran MH, Unger RH, Recant L, Polk HC, Kilo C, Levin ME (1966). A glucagon-secreting alpha-cell carcinoma of the pancreas. *The New England Journal of Medicine*.

[3] Wilkinson DS (1971). Necrolytic migratory erythema with pancreatic carcinoma. *Proceedings of the Royal Society of Medicine*.

[4] Mallinson CN, Bloom SR, Warin AP, Salmon PR, Cox B (1974). A glucagonoma syndrome. *The Lancet*.

[5] van Beek AP, de Haas ERM, van Vloten WA, Lips CJM, Roijers JFM, Canninga-van Dijk MR (2004). The glucagonoma syndrome and necrolytic migratory erythema: a clinical review. *European Journal of Endocrinology*.

[6] Wermers RA, Fatourechi V, Kvols LK (1996). Clinical spectrum of hyperglucagonemia associated with malignant neuroendocrine tumors. *Mayo Clinic Proceedings*.

[7] Kheir SM, Omura EF, Grizzle WE (1986). Histologic variation in the skin lesions of the glucagonoma syndrome. *American Journal of Surgical Pathology*.

[8] Johnson SM, Smoller BR, Lamps LW, Horn TD (2003). Necrolytic migratory erythema as the only presenting sign of a glucagonoma. *Journal of the American Academy of Dermatology*.

[9] Tanaka S, Yamasaki S, Matsushita H (2000). Duodenal somatostatinoma: a case report and review of 31 cases with special reference to the relationship between tumor size and metastasis. *Pathology International*.

[10] Altimari AF, Bhoopalam N, O’Dorsio T (1986). Use of a somatostatin analog (SMS 201–995) in the glucagonoma syndrome. *Surgery*.

